# High-Resolution Nerve Ultrasound Abnormalities in POEMS Syndrome—A Comparative Study

**DOI:** 10.3390/diagnostics11020264

**Published:** 2021-02-09

**Authors:** Marc Dörner, Mihai Ceanga, Frank Schreiber, Jan-Hendrik Stahl, Cornelius Kronlage, Julia Wittlinger, Magdalena Kramer, Sophia Willikens, Stefanie Schreiber, Alexander Grimm, Natalie Winter

**Affiliations:** 1 Center for Neurology, Tuebingen University Hospital and Hertie-Institute for Clinical Brain Research, Eberhard Karls University Tuebingen, 72076 Tuebingen, Germany; jan-hendrik.stahl@med.uni-tuebingen.de (J.-H.S.); Cornelius.kronlage@med.uni-tuebingen.de (C.K.); julia.wittlinger@med.uni-tuebingen.de (J.W.); magdalena.kramer@med.uni-tuebingen.de (M.K.); sophia.willikens@med.uni-tuebingen.de (S.W.); alexander.grimm@med.uni-tuebingen.de (A.G.); natalie.winter@med.uni-tuebingen.de (N.W.); 2 Department of Forensic Psychiatry, University Hospital of Psychiatry Zurich, University of Zurich, 8008 Zurich, Switzerland; 3 Hans Berger Department of Neurology, Jena University Hospital, Friedrich Schiller University Jena, 07740 Jena, Germany; Mihai.Ceanga@med.uni-jena.de; 4 Department of Neurology, Otto-von-Guericke University, 39120 Magdeburg, Germany; Frank.schreiber@dzne.de (F.S.); stefanie.schreiber@med.ovgu.de (S.S.); 5 German Center for Neurodegenerative Diseases (DZNE) within the Helmholtz Association, 39120 Magdeburg, Germany; 6 Center for Behavioural Brain Sciences (CBBS), 39120 Magdeburg, Germany

**Keywords:** peripheral nerve imaging, high-resolution nerve ultrasound, POEMS syndrome, CIDP, immune-mediated neuropathies

## Abstract

Background: High-resolution nerve ultrasound (HRUS) has been proven to be a valuable tool in the diagnosis of immune-mediated neuropathies, such as chronic inflammatory demyelinating polyradiculoneuropathy (CIDP). POEMS syndrome (polyneuropathy, organomegaly, endocrinopathy, M-protein, skin changes) is an important differential diagnosis of CIDP. Until now, there have been no studies that could identify specific HRUS abnormalities in POEMS syndrome patients. Thus, the aim of this study was to assess possible changes and compare findings with CIDP patients. Methods: We retrospectively analyzed HRUS findings in three POEMS syndrome and ten CIDP patients by evaluating cross-sectional nerve area (CSA), echogenicity and additionally calculating ultrasound pattern scores (UPSA, UPSB, UPSC and UPSS) and homogeneity scores (HS). Results: CIDP patients showed greater CSA enlargement and higher UPSS (median 14 vs. 11), UPSA (median 11.5 vs. 8) and HS (median 5 vs. 3) compared with POEMS syndrome patients. However, every POEMS syndrome patient illustrated enlarged nerves exceeding reference values, which were not restricted to entrapment sites. In CIDP and POEMS syndrome, heterogeneous enlargement patterns could be identified, such as inhomogeneous, homogeneous and regional nerve enlargement. HRUS in CIDP patients visualized both increased and decreased echointensity, while POEMS syndrome patients pictured hypoechoic nerves with hyperechoic intraneural connective tissue. ***Discussion:*** This is the first study to demonstrate HRUS abnormalities in POEMS syndrome outside of common entrapment sites. Although nerve enlargement was more prominent in CIDP, POEMS syndrome patients revealed distinct echogenicity patterns, which might aid in its differentiation from CIDP. Future studies should consider HRUS and its possible role in determining diagnosis, prognosis and treatment response in POEMS syndrome.

## 1. Introduction

High-resolution nerve ultrasound (HRUS) has been proven to be a valuable tool in the diagnosis of peripheral nervous system diseases such as immune-mediated neuropathies or central nervous system diseases such as amyotrophic lateral sclerosis, which also affects peripheral nerves [1,2,3]. The detection rate of immune-mediated neuropathies can be even improved by 20% if HRUS is added to conventional nerve conduction studies and clinical examination [4].

Chronic inflammatory demyelinating polyradiculoneuropathy (CIDP) belongs to the group of immune-mediated neuropathies and is associated with segmental or diffuse nerve enlargement (with a predominance in proximal nerve segments), i.e., increased cross sectional nerve area (CSA), greater nerve vascularization and fascicular enlargement in HRUS [5].

An important differential diagnosis of CIDP is POEMS syndrome (polyneuropathy, organomegaly, endocrinopathy, M-protein, skin changes), a rare variant of multiple myeloma (MM), which requires the existence of a polyneuropathy and a monoclonal plasma-cell proliferative disorder for the diagnosis. Elevated serum levels of vascular endothelial growth factor (VEGF) are also among the diagnostic criteria and can be used as a marker for therapeutic response. In its clinical presentation, the predominantly demyelinating polyneuropathy mimics CIDP with progressive sensorimotor deficits in both proximal and distal nerve segments. Moreover, polyneuropathy presents often as the initial and isolated symptom in POEMS syndrome and is mostly symmetric, distal, painful and rapidly progressive. Since treatment is different from CIDP, an accurate diagnosis is important [6].

Until now, there have been only two studies taking account of HRUS abnormalities in POEMS syndrome. In the first study, eight POEMS syndrome patients underwent HRUS evaluation for possible changes in CSA, fascicle size and echogenicity of the median, ulnar, tibial and peroneal nerves. However, no specific HRUS abnormalities could be identified [7]. In the same year, a case report of a POEMS syndrome patient described diffusely thickened peripheral nerves of both upper limbs with increased arterial blood flow in ultrasound imaging [8]. The second study observed HRUS alterations in the median nerve of 33 POEMS syndrome patients. HRUS of these patients showed greater CSA than in healthy individuals, but still CSA did not exceed reference values [9].

Thus, the aim of our study was to assess possible HRUS pathologies in patients with POEMS syndrome and compare them with findings in CIDP patients by using some novel approaches. In contrast to the already exisiting studies, we utilized newly established CSA normal values with more measuring sites. Additionally, we analyzed the enlargement patterns of the examined nerves and calculated different quantitative scores to evaluate peripheral nerve pathologies, which might possibly aid in its differentiation from CIDP. Further, we evaluated the overall echointensity of several nerves and compared them according to known classifications.

## 2. Methods

In this study we retrospectively included three patients who were diagnosed with POEMS syndrome according to the current diagnostic criteria ([10]; three men, mean age at onset 65.0 ± 6.1 years, range 58–69 years). We compared HRUS findings with ten patients who met the EFNS diagnostic criteria for definite CIDP ([11]; four men and six women, mean age at onset 56.1 ± 11.4 years, range 37–73 years). HRUS in POEMS syndrome patients was performed before treatment initiation. Since the majority of our CIDP patient sample was diagnosed years before our POEMS patients and HRUS was not always available then, HRUS in CIDP patients mainly took place after treatment onset. All patients were recruited from the Center of Neurology at the University of Tuebingen except for one POEMS syndrome patient (Department of Neurology at the University of Jena) and gave their written consent for participation in the study. Table 1 and Table 2 present the characteristics of the patients.

HRUS examinations were performed by different neurologists using 14–18 MHz broadband ultrasound probes (Mindray TE7, Darmstadt, Germany) and took approximately 40 min for each patient. In one POEMS syndrome patient (#3; Table 1) and in one CIDP patient (#8; Table 2), another ultrasound system was used (#3: 14 MHz broadband ultrasound probe, Aplio 400, Toshiba medical systems, Japan; #8: 24 MHz broadband ultrasound probe, Canon Aplio i800, Neuss, Germany). To avoid anisotropy, the ultrasound probe was kept perpendicular to the nerves. Moreover, to avert any artificial nerve deformity, the extremities were maintained in the neutral position and only the weight of the ultrasound probe was applied to the measurement points. HRUS measurements were repeated with intraclass coefficient correlation >90% (intra-and interobserver accuracy >90% [12]).

**Figure 1:**
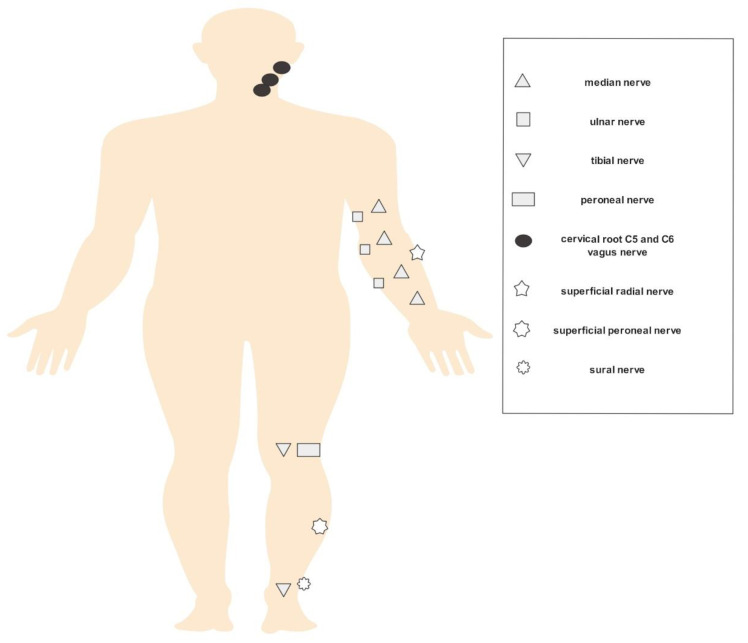
HRUS (high-resolution nerve ultrasound) measurement points.

The evaluation for each patient was conducted according to the well-established ultrasound pattern sum score (=UPSS), which includes sensorimotor nerves (median, ulnar, tibial and peroneal nerves: ultrasound pattern score A (= UPSA)), cervical roots (C5 and C6) and vagal nerves (ultrasound pattern score B = UPSB), as well as sensory nerves (sural, superficial radial and superficial peroneal nerves: ultrasound pattern score C (= UPSC)). Measurements took place at predefined sites and comprehended CSA within the hyperechoic epineural rim for all nerves (axial plane) and diameter for C5 and C6 (longitudinal plane). The median and ulnar nerves were scanned at the wrist, at the mid-forearm, at the elbow and at the mid-upper arm. The tibial and peroneal nerves were measured at the popliteal fossa, and the tibial (besides the sural) nerves were additionally analyzed at the ankle. Furthermore, the radial superficial nerve was examined at the forearm, the peroneal superficial nerve at mid-calf level and the vagus nerve close to the carotid artery at bifurcation level. Diameters of the cervical roots 5 and 6 were assessed after exiting the intervertebral foramen [12,13]. Due to the symmetrical involvement of the polyneuropathy, most measurements were performed on the right side (in some cases on the left side as well). Figure 1 demonstrates the measurement points of the examined nerves.

Regarding the UPSA, one point is given for every nerve enlargement >100% and <150%, and two points for each enlargement >150% of the upper normal values (UPSA maximum 16 points). For the UPSB and UPSC, each nerve exceeding normal values is rated with one point (UPSB and UPSC maximum three points, respectively). Thus, the UPSS as a total score can reach up to 22 points [12].

Additionally, homogeneity scores (=HS) for each patient were obtained. Homogeneity patterns are categorized into normal (no enlargement), regional enlargement (enlarged and normal values in the same nerve), inhomogeneous enlargement (generalized CSA enlargement in a nerve, enlargement >150% and <150% above normal limit in the same nerve), mild homogeneous enlargement (generalized nerve enlargement in a nerve <150% above normal limit) and overt homogeneous enlargement (generalized nerve enlargement in a nerve >150% above normal limit). For scoring, −1 point is given for regional enlarged nerves, 0 points for no enlargement, 1 point for inhomogeneous, 2 points for mild homogeneous and 3 points for overt homogeneous enlargement. For analysis, the median, ulnar and tibial nerves are examined and a total score of 9 points can be reached for each patient (range −3–9 points [12,14]).

Echogenicity of the nerves was rated according to Padua et al. [15] and divided into class 1 (large nerves with hypoechoic nerves/fascicles), class 2 (large nerves with heterogeneous hyper- and hypoechoic fascicles) and class 3 (normal size nerve with hyperechoic fascicles). Finally, we evaluated the intranerve CSA ratios (maximal CSA/minimal CSA without entrapment sites) as well as the entrapment site CSA ratios (CSA median nerve wrist/CSA median nerve forearm, and CSA ulnar nerve elbow/CSA ulnar nerve upper arm) for the median and ulnar nerves.

For each examined nerve (CSA and diameter) as well as for the UPSS and HS, median values of the POEMS patient group and the CIDP patient group were calculated and compared with each other. When measurements took place on both sides, the most involved nerve was used (see also [14]).

Alongside HRUS examinations, nerve conduction studies (NCS) were performed according to existing literature [11].

## 3. Results

HRUS showed abnormalities in both POEMS and CIDP patients for almost all examined nerves and were not restricted to entrapment sites. Table 3 gives an overview on the examined nerves.

The findings from the upper extremitiy nerves are summarized in Table 4 and Table 5. Both patient groups visualized nerve pathologies along the whole course of the median and ulnar nerves, but CIDP patients tended to have slightly higher CSA values compared with POEMS syndrome patients.

**Figure 2:**
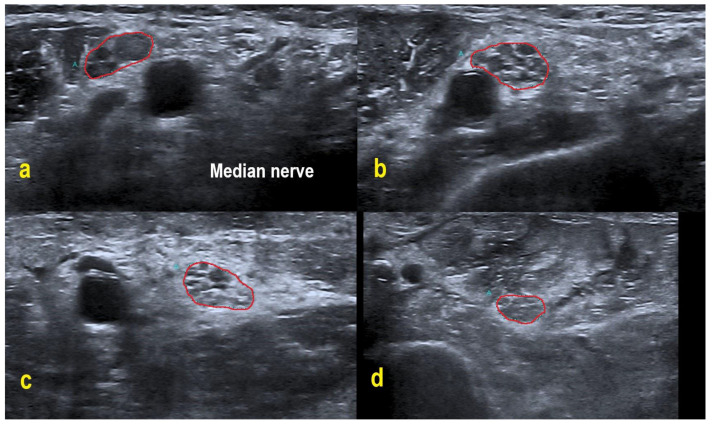
HRUS in POEMS syndrome patient #3. HRUS demonstrated overt homogeneous enlargement of the CSA in the right median nerve (within red border; CSA up to 2.0-fold increased). Axilla (**a**): CSA 20 mm². Upper arm (**b**): 25 mm². Midarm (**c**): 23 mm². Forearm (**d**): 15 mm². Of note, the intraneural echointensity reflects hyperechoic interfascicular tissue next to hypoechoic fascicles.

POEMS patients demonstrated overt homogeneous (two patients) and regional enlargement (one patient) in the median nerve, while CIDP patients had overt homogeneous (five patients), inhomogeneous (two patients), regional (two patients) and mild homogeneous (one patient) enlargement patterns. HRUS findings of the right median nerve in POEMS syndrome patient #3 are shown in Figure 2. Enlargement patterns in the ulnar nerve were equally heterogeneous. Two POEMS syndrome patients exhibited inhomogeneous and another mild homogeneous enlargement, five CIDP patients had overt homogeneous, two inhomogeneous, one mild homogeneous and one regional enlargement (and also one patient without an enlargement pattern).

The median value for the intranerve CSA ratio in the median nerve was 1.6 in POEMS syndrome vs. 1.55 in CIDP, and for the ulnar nerve it was 1.1 vs. 1.2. Concerning the entrapment site CSA ratio of the median nerve, the median value was 1.1 in POEMS syndrome vs. 1.2 in CIDP. However, POEMS syndrome patients depicted obviously higher entrapment site CSA ratios in the ulnar nerve in comparison to CIDP patients (median 1.5 vs. 0.8).

In contrast to the median and ulnar nerves, POEMS syndrome patients displayed less obvious CSA enlargements in the nerves of the lower extremities (Table 6). Nerve enlargement was almost only restricted to proximal nerve segments of the tibial nerve. CIDP patients had obviously higher CSA values in the peroneal nerve and distal segments of the tibial nerve as compared to POEMS patients.

The enlargement patterns in the tibial nerve showed regional enlargement in two POEMS syndrome and two CIDP patients. Four CIDP patients had inhomogeneous, two mild homogeneous and one overt homogeneous nerve enlargement. One POEMS syndrome and one CIDP patient illustrated normal tibial nerves.

As demonstrated for the CSA values of the sensorimotor nerves, diameters of C5 and C6 were more increased in CIDP patients than in POEMS syndrome. Still, CIDP patients showed greater heterogeneity among the nerves (internerve variability). In every POEMS syndrome patient, diameters of C5 and C6 and CSA of the vagus nerve were enlarged, while seven out of ten CIDP patients had enlargement of C5, five out of nine enlargement of C6 and six out of ten enlargement of the vagus nerve (Table 7).

Concerning the pure sensory nerves, every POEMS syndrome patient as well as seven out of nine CIDP patients displayed CSA enlargements of the sural nerve. However, again greater internerve variability could be identified in the remaining nerves. Only in one POEMS syndrome patient and only in four out of nine CIDP patients could enlargement of the superficial radial nerve be observed. The same applies to the superficial peroneal nerve with no POEMS syndrome patient and only five out of nine CIDP patients having CSA enlargement (Table 8).

Corresponding to CSA values, quantitative scores in CIDP patients tended to be higher than in POEMS syndrome, although differences were again not prominent (median UPSA in CIDP 11.5 vs. 8 points in POEMS syndrome, median UPSB 1.5 vs. 3, median UPSC 1 vs. 1, median UPSS 14 vs. 11 and median HS 5 vs. 3; Table 9).

**Figure 3:**
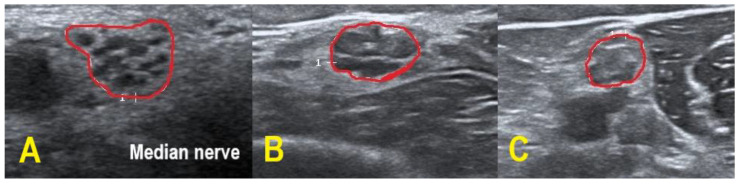
HRUS of the right upper arm median nerve. POEMS syndrome patient #1 demonstrated hypoechoic fascicles and hyperechoic intraneural connective tissue ((**A**) within red border). In CIDP patient #6, HRUS depicted hypoechoic next to hyperechoic fascicles ((**B**) within red border), whereas HRUS in CIDP patient #9 showed hyperechoic fascicles ((**C**) within red border).

Four CIDP patients and every POEMS syndrome patient showed hypoechoic nerves (class 1), whereas the remaining CIDP patients demonstrated a more heterogeneous pattern with hypo- and hyperechoic fascicles (class 2). No patient exhibited class 3 pattern. In contrast to the patients with CIDP, the examined POEMS syndrome patients featured additional hyperechoic intraneural connective tissue. Figure 3 exemplifies the different HRUS findings.

NCS in every POEMS syndrome patient revealed a demyelinating sensorimotor polyneuropathy with secondary axonal degeneration (reduced conduction velocity (CV) and compound muscle action potential (CMAP), prolonged distal motor latency in the upper extremities; severe axonal involvement in the lower extremities with loss of CMAP and sensory nerve action potential, plus not recordable CV). Similar to POEMS patients, every CIDP patient illustrated a predominantly demyelinating sensorimotor involvement and secondary axonal nerve damage.

## 4. Discussion

This is the first study that shows HRUS pathologies in POEMS syndrome outside of common entrapment sites [7]. CSA enlargement occurred in both CIDP and POEMS syndrome patients (as compared to normative values) and could be further quantified and categorized by using the UPSS and HS.

Overall, CSA enlargement was slightly more prominent in CIDP patients than in POEMS syndrome. However, significances have not been calculated due to the small sample size. Concerning the entrapment site CSA ratios, our POEMS patients partly portrayed even obviously higher ratios compared with CIDP. As reported before in several studies, CIDP patients demonstrated nerve enlargement with a predominance in proximal nerve segments [1,5]. Plus, CIDP patients illustrated heterogeneous morphologies, having regional, inhomogeneous and homogeneous nerve enlargement with increased or decreased echointensity, as already described in the past [14,15]. In line with this, our POEMS syndrome patients had heterogeneous nerve enlargement patterns. Proximal nerve segments were also more frequently enlarged than distal nerve segments, but, different to our CIDP patients, in POEMS syndrome, the upper extremities pictured obvious greater nerve enlargement than the lower extremities. Interestingly, clinical findings in our POEMS syndrome patients identified greater involvement of the lower limbs, which has also been presented by Nasu et al. [19]. It might be possible that nerve pathology in lower limb nerves could not be adequately seen by HRUS, as more proximal regions as the sciatic nerves have not been scanned. Electrophysiological findings in our POEMS patients are in line with past studies, and discrimination between POEMS syndrome and CIDP might be difficult when bearing in mind nerve conduction studies alone [7,19].

According to Grimm et al., a UPSA ≥ 7 points or a UPSS ≥ 10 points is suggestive of CIDP, which is consistent to our findings in CIDP patients [20]. Although the UPSS tended to be higher in CIDP, patients with POEMS syndrome also exceeded these thresholds. The same holds true for the HS in our POEMS syndrome patients, which were similar to CIDP patients in past studies [14]. Considering these results, quantitative scores such as the UPSS and HS might not be sufficient to discriminate between these diseases.

Therefore, the observed echogenicity patterns in our POEMS patients could be a promising finding. Lucchetta et al. [7] already described hypoechogenic nerves in POEMS syndrome, as was the case in our study. Our POEMS patients presented additional hyperechoic intraneural connective tissue, which is an interesting morphological observation and not known to appear in CIDP. Furthermore, hypoechoic nerves are associated with better treatment response in CIDP (possibly reflecting active inflammation) and can be used as a prognostic marker, while in clinically progressive CIDP, hyperechoic nerves are frequently found (possibly reflecting axonal degeneration [18,21,22]). Follow-up studies have to prove if these findings are applicable to POEMS syndrome patients. The histological correlate behind the increased and hyperechoic interfascicular tissue has to be clarified by histopathological studies in future projects.

We must consider that in the case of MM, several neuropathy types can occur: mainly axonal neuropathies caused by the disease itself or by chemotherapy used for the disease. However, the description of hypoechoic nerve swelling in POEMS is of note. If hypo- or hyperechoic nerve swelling is seen in MM patients, examiners must be aware of extraossar infiltration of plasmocytoma in the nerve, which is overall quite rare. Second, paraproteinemic neuropathies—next to MM—might present like CIDP, and thus, might offer the same heterogeneous echointensities as the latter. Then, therapeutic steps are the same as in classical CIDP [23,24,25].

Differential diagnosis between CIDP and POEMS syndrome remains challenging. Besides clinical examination, nerve conduction studies and HRUS, other options should be considered in the diagnostic work up in the future. For example, magnetic resonance imaging (MRI) of the brain and spine could be used to further aid in the diagnosis. Ziff et al. identified pachymeningeal thickening in POEMS syndrome patients in contrast to patients with CIDP [26]. Combining HRUS and MRI might help to achieve even greater diagnostic accuracy, but future systematic studies are needed to prove this hypothesis [27]. MRI, especially diffusion tensor imaging (DTI) based tractography, might be used in the future to verify measurements by HRUS. DTI based tractography allows us to visualize and conclude about the course of nerve tracts and is able to differentiate tissues on the basis of diffusion anisotropy differences. This imaging method has already exemplified in the past its potential value (e.g., characterize changes associated with age and neurodegenerative diseases). Therefore, by analyzing changes in parameters such as Fractional Anisotropy, it might be possible to additionally infer on the state of the examined nerves [28,29,30]. To obtain sufficient precision of the research, it will be important to use suitable methods to eliminate spatial systematic errors [31,32].

There are some limitations of the study. While there were similar HRUS findings in POEMS syndrome in the mentioned previous study (hypoechogenic nerves [7]), we also report new findings (general CSA enlargement exceeding reference values, hyperechoic intraneural connective tissue) in a much smaller patient sample. Thus, our findings have to be treated with caution and need confirmation in future studies. Secondly, we did not examine nerve vascularity or abnormalities of single fascicles, which might have helped in the differentiation between CIDP and POEMS syndrome. Additionally, we have to critically point out that HRUS in our CIDP patients was partly performed before and partly after treatment onset, while HRUS in POEMS syndrome took place before treatment initiation, which further restricts comparison of the two patient groups. Nerve enlargement may vary with disease duration and treatment. Some studies found that longer disease duration or longer intervals between symptom onset and treatment in CIDP patients is associated with larger nerve size [33,34]. Consequently, we cannot rule out that our findings are biased to a certain degree. However, they depict daily routine in outpatient hospitals as diagnosis is often delayed in CIDP as well as in POEMS syndrome and treatment had already begun. Despite the fact that these results cannot be necessarily applied to POEMS syndrome, future studies should consider this and include more treatment naïve patients to evaluate the role of HRUS in determining prognosis and treatment efficacy in POEMS syndrome. At last, it should be mentioned that the position of the ultrasound probe, particularly the arm angulation, is an important factor on the assessment of the CSA. Since examinations were performed by different neurologists, we have to take into consideration the subjectivity concerning the positioning of the probe.

## 5. Conclusions

Demonstrating for the first time specific HRUS nerve abnormalities in POEMS syndrome is an important finding of this study, since HRUS has already proven in the past to be a helpful tool in the diagnosis and monitoring of disease course of peripheral nervous system diseases [35]. Particularly, the discovery of distinct echogenicity patterns in POEMS syndrome might aid in its differentiation from CIDP. Future follow-up studies have to analyze if HRUS could not only serve as a door opener in immune-mediated neuropathies such as CIDP, but also in POEMS syndrome, and should focus on the addressed issues.

## Figures and Tables

**Table 1:** Characteristics of patients with POEMS syndrome (polyneuropathy, organomegaly, endocrinopathy, M-protein, skin changes).

Pt. #	Onset Age/Gender	Disease Onset	Neuropathy Symptoms and Signs	Major Criteria POEMS	Minor Criteria POEMS	M Protein	Highest VEGF
1	58/m	11/2016	Distal symmetrical hypaesthesias and paraparesis (LE), gait ataxia	Multiple osteosclerotic bone lesions, Castleman’s disease	Hepatosplenomegaly, oedema, skin changes	IgM lambda	/
2	68/m	11/2016	Distal symmetrical hypaesthesias (LE), no paresis, gait ataxia	Osteosclerotic and osteolytic bone lesions, elevated VEGF	Hepatosplenomegaly, syndrome of inappropriate antidiuretic hormone secretion (SIADH)	IgG lambda	668 pg/mL (<100 pg/mL)
3	69/m	05/2016	Distal symmetrical hypaesthesias and tetraparesis (LE > UE), gait ataxia, neuropathic pain in both feet	Osteolytic bone lesions, elevated VEGF	hepatomegaly, hypergonadotropic hypogonadism, hypothyroidism, oedema	IgG lambda	859 pg/mL (<100 pg/mL)

Abbreviations: m: male, LE: lower extremity, UE: upper extremity.

**Table 2:** Characteristics of patients with CIDP (chronic inflammatory demyelinating polyradiculoneuropathy).

Pt. #	Onset Age/Gender	Disease Onset	Neuropathy Symptoms and Signs	Therapy
1	55/m	02/2009	Distal symmetrical hypaesthesias (LE) and neuropathic pain in both feet	Intravenous immunoglobulins (IVIG)
2	65/f	2011	Distal symmetrical hypaesthesias (LE + UE) and tetraparesis, neuropathic pain in both hands and LE	Methylprednisolone
3	62/f	2013	Distal symmetrical hypaesthesias and paraesthesias of both feet and hands and tetraparesis	Methylprednisolone and IVIG
4	37/f	1990	Distal symmetrical paraesthesias of both hand and feet, tetraparesis	Prednisolone, Azathioprine, IVIG, Mycophenolate mofetil
5	73/f	2012	Distal symmetrical hypaesthesias (LE + UE) and tetraparesis, gait ataxia, neuropathic pain	IVIG, Rituximab
6	44/m	2004	Distal symmetrical hypaesthesias and paraesthesias (LE + UE), paraparesis of LE, neuropathic pain of both feet and hands	Prednisolone, Cyclophosphamide, IVIG, Rituximab
7	44/f	2004	Distal symmetrical hypaesthesias of UE, tetraparesis	Prednisolone, Azathioprine, Ciclosporin, IVIG, Rituximab
8	57/m	2004	Distal symmetrical hypaesthesias and paraesthesias (LE + UE), no paresis	Prednisolone, Azathioprine, plasmapheresis, IVIG, Methylprednisolone
9	67/m	2010	Distal symmetrical hypaesthesias and paraesthesias (LE + UE), no paresis, gait ataxia	IVIG
10	57/f	06/2017	Distal symmetrical hypaesthesias (LE + UE) and tetraparesis, neuropathic pain of LE	Prednisolone, IVIG, plasmapheresis

Abbreviations: m: male, f: female, LE: lower extremity, UE: upper extremity.

**Table 3:** HRUS median values.

CSA (Cross Sectional Nerve area) in mm^2^, Diameter C5–6 in mm	POEMS	CIDP	Normative Values *
	**Median (Range)**		
Median nerve wrist	**16 (16–17)**	**16.5 (8–33)**	<13 **
Median nerve forearm	**15 (9–21)**	**18 (8–69)**	<10
Median nerve midarm	**25 (10–34)**	**20 (10–45)**	<12.5
Median nerve upper arm	**25 (12–29)**	**27 (12–87)**	<12
Ulnar nerve forearm	**12 (10–16)**	**16 (8–59)**	<8.5
Ulnar nerve midarm	**22 (12–41)**	**13 (9–32)**	<9–10 ***
Ulnar nerve upper arm	**14 (11–14)**	**16.5 (9–86)**	<9.5
Tibial nerve popliteal	**55 (22–57)**	**45 (24–65)**	<33 (<37 for men > 60 years)
Tibial nerve distal	11 (10–12)	**18 (8–33)**	<14
Peroneal nerve proximal	11 (9–13)	**15 (12–54)**	<11.5
C5	**3.2 (3.0–3.7)**	**3.5 (2.2–8.9)**	<2.9
C6	**4.3 (4.3–5.4)**	**4.9 (3.1–11.2)**	<4.2
Vagus nerve	**5**	**4 (2–8)**	<3.5
Sural nerve	**5 (5–6)**	**4 (2–6)**	<3.5
Superficial radial nerve	**3 (2–4)**	2.4 (2–5)	<3
Superficial peroneal nerve	2.5 (2–3)	**4 (1–21)**	<3.5

Note: Pathological values are marked bold. * [12]. ** [16]. *** [17].

**Table 4:** HRUS findings in upper limb nerves: median nerve CSA values in mm^2^.

	CSA at Wrist		CSA at Forearm		CSA at Midarm		CSA at Upper Arm	
Pt. #	Right	Left	Right	Left	Right	Left	Right	Left
POEMS								
1	**16**		**21**		**34**		**29**	
2	**17**		9		10		**12**	
3		**16**	**15**	**14**	**23**	**25**	**25**	**22**
CIDP								
1	**17**		**14**		**16**		**15**	
2	**16**	**16**	**17**	**18**	10	**18**	**12**	**24**
3	**15**		8		**15**		**16**	
4	**20**		**69**		**45**		**87**	
5	**15**		**23**		**27**		**18**	
6	**28**		**23**		**27**		**37**	
7	**24**		**18**		**22**		**35**	
8	**33**		**24**		**23**		**30**	
9	8		**11**		**16**		**35**	
10	**13**		8		10		**12**	

Note: Pathological values are marked bold. CSA normal values: at wrist < 13 mm^2^ [16]; at forearm < 10 mm^2^; at midarm < 12.5 mm^2^; at upper arm < 12 mm^2^ [12].

**Table 5:** HRUS findings in upper limb nerves: ulnar nerve CSA values in mm^2^.

	CSA at Forearm		CSA at Midarm		CSA at Upper Arm	
Pt. #	Right	Left	Right	Left	Right	Left
POEMS						
1	**16**		**22**		**14**	
2	**10**		**12**		**11**	
3	**10**	**12**	**20**	**41**	**10**	**14**
CIDP						
1	**15**		**17**		**12**	
2	**15**	**17**	8	**13**	**11**	**16**
3	8		**13**		9	
4	**59**		**12**		**86**	
5	**18**		**15**		**15**	
6	**23**		**28**		**45**	
7	**10**		**11**		**17**	
8	**20**		**32**		**23**	
9	8		**10**		**22**	
10	**12**		9		**11**	

Note: Pathological values are marked bold. CSA normal values: at midarm < 9–10 mm^2^ [17]; at forearm < 8.5 mm^2^; at upper arm < 9.5 mm^2^ [12].

**Table 6:** HRUS findings in lower limb nerves: tibial and peroneal nerve CSA values in mm^2^.

	Tibial Nerve Popliteal		Tibial Nerve Distal		Peroneal Nerve Proximal	
Pt. #	Right	Left	Right	Left	Right	Left
POEMS						
1	22		11		11	
2	57 *		12		**13**	
3	55 *	29	8	10	9	9
CIDP						
1	**45**		**25**		**16**	
2	**65**	**40–65**	13		**15**	**17**
3	**43**		8		**15**	
4	**57**		**18**		**54**	
5	**63**		**18**		**14**	
6	**65**		**33**		**51**	
7	**34**		**15**		**14**	
8	24		10		**14**	
9	**41**		**26**		**15**	
10	**45**		**18**		**12**	

Note: Pathological values are marked bold. CSA normal values: tibial nerve popliteal < 33 mm^2^ (* < 37 mm^2^ for men > 60 years at time of examination); tibial nerve distal < 14 mm^2^; peroneal nerve proximal < 11.5 mm^2^ [12].

**Table 7:** HRUS findings in cervical roots (diameter in mm) and vagus nerve CSA values in mm^2^.

	Diameter of C5		Diameter of C6		CSA of Vagus Nerve
Pt. #	Right	Left	Right	Left	Right
POEMS					
1	**3.2**		**5.4**		**5**
2	**3.0**		**4.3**		**5**
3		**3.7**		**4.3**	
CIDP					
1	**3.2**		4.0		3
2	2.8		3.1		2
3	**3.2**		3.9		3
4	**8.9**		**11.2**		**5**
5	2.8		3.9		**4**
6	**6.1**		**8.8**		**8**
7	**4.3**		**5.5**		**6**
8	**3.8**		**4.9**		2
9	**7.0**		**7.0**		**4**
10	2.2				**4**

Note: Pathological values are marked bold. CSA normal values: diameter of C5 < 2.9 mm; diameter of C6 < 4.2 mm; vagus nerve < 3.5 mm^2^ [12].

**Table 8:** HRUS findings in sensory nerves: sural, superf. radial and superf. peroneal nerves in mm^2^.

	CSA of Sural Nerve	CSA of Superf. Radial Nerve	CSA of Superf. Peroneal Nerve
Pt. #	Right	Right	Right
POEMS			
1	**5**	**4**	2
2	**5**	2	3
3	**6**		
CIDP			
1	**4**		
2	**4**	2	3
3	3	**4**	1
4	**6**	**3**	**14**
5	**6**	2	**7**
6	**5**	**5**	**7**
7	**4**	**5**	**4**
8		2	**21**
9	**3.7**	2.4	3
10	2	2	3

Note: Pathological values are marked bold. CSA normal values: sural nerve < 3.5 mm^2^; superficial radial nerve < 3.0 mm^2^; superficial peroneal nerve < 3.5 mm^2^ [12].

**Table 9:** UPSA, UPSB, UPSC, UPSS, HS and echogenicity classes for POEMS and CIDP patients.

Pt. #	UPSA	UPSB	UPSC	UPSS	HS	Echogenicity Class *
POEMS						
1	9	3	2	14	4	1
2	6	3	1	10	−1	1
3	8	2 ^a^	1 ^a^	11 ^a^	3	1
CIDP						
1	10	1	1 ^b^	12 ^b^	4	2
2	12	0	1	13	3	1
3	4	1	1	6	−2	2
4	15	3	3	21	7	2
5	14	1	2	17	7	1
6	16	3	3	22	9	1
7	12	3	3	18	6	1
8	11	2	1 ^c^	14 ^c^	6	2
9	10	3	1	14	1	2
10	6	1 ^d^	0	7 ^d^	3	2

Note: UPSA: peripheral sensorimotor nerves range 0–16; UPSB: cervical roots and vagus nerve range 0–3; UPSC: sensory nerves range 0–3; UPSS: total score range 0–22. HS: homogeneity score range −3–9. * According to [15,18]. ^a^ UPSB range 0–2, UPSC range 0–1, UPSS range 0–19. ^b^ UPSC range 0–1, UPSS range 0–20. ^c^ UPSC range 0–2, UPSS range 0–21. ^d^ UPSB range 0–2, UPSS range 0–21.

## Data Availability

The data presented in this study are available on request from the corresponding author.
